# Temperature induced changes in the optical properties of skin in vivo

**DOI:** 10.1038/s41598-020-80254-9

**Published:** 2021-01-12

**Authors:** Tyler W. Iorizzo, Peter R. Jermain, Elena Salomatina, Alona Muzikansky, Anna N. Yaroslavsky

**Affiliations:** 1 Advanced Biophotonics Laboratory, University of Massachusetts Lowell, 175 Cabot Street, Lowell, MA 01854 USA; 2 Department of Dermatology, Massachusetts General Hospital, 50 Staniford Street, Boston, MA 02114 USA; 3 Biostatistics Center, Massachusetts General Hospital, Boston, MA 02114 USA

**Keywords:** Spectrophotometry, Biophotonics

## Abstract

Knowledge of temperature-induced changes of skin optical properties is required for accurate dosimetry of photothermal treatments. We determined and compared in vivo optical properties of mouse ear skin at different temperatures. The diffuse reflectance, total and diffuse transmittance were measured in the spectral range from 400 to 1650 nm using an integrating sphere spectrometer at the temperatures of 25 °C, 36 °C and 60 °C. Target temperatures were attained and maintained using an automated heater equipped with a sensor for feed-back and control. Temperature and temperature induced morphological changes of skin were monitored using an infrared thermal camera and reflectance confocal microscopy, respectively. An inverse Monte Carlo technique was utilized to determine absorption, scattering, and anisotropy factors from the measured quantities. Our results indicate significant differences between the optical properties of skin at different temperatures. Absorption and scattering coefficients increased, whereas anisotropy factors decreased with increasing temperature. Changes in absorption coefficients indicate deoxygenation of hemoglobin, and a blue shift of water absorption bands. Confocal imaging confirmed that our observations can be explained by temperature induced protein denaturation and blood coagulation. Monitoring spectral responses of treated tissue may become a valuable tool for accurate dosimetry of light treatments.

## Introduction

Therapeutic and surgical applications of light often cause an increase in temperature^1–13^, which may alter the optical properties of target and adjacent organs. Such changes have been reported in several tissues, including adipose^14^, brain^15,16^, skin^17–19^, prostate^20^, and liver^21–23^. Thus, there is a great need to examine dependence of the optical properties on tissue temperature. Several groups investigated the impact of freezing or heating on the ex vivo optical properties^14,17,19–23^. In vivo studies are rare and often restricted to narrow wavelength ranges^24–27^. Yet, quantifying temperature induced changes in optical properties of in vivo tissue would provide a better understanding of tissue response during phototherapy and would ultimately lead to the improvement of light dosimetry. Previously, we have used a mouse ear skin model to examine the differences in the optical constants measured in vivo and ex vivo^19^. The goal of this study was to investigate temperature dependence of the optical properties of skin, in vivo, using integrating sphere measurements combined with inverse Monte Carlo technique, and to monitor heat induced morphological changes to the tissue structure using reflectance confocal microscopy.

## Results

We have determined in vivo absorption coefficients, scattering coefficients, and anisotropy factors of mouse ear tissues at three temperatures of 25 °C, 36 °C, and 60 °C. In total, thirty-two sets of data were acquired, processed, and analyzed, including twelve sets at 25 °C, ten sets at 36 °C, and ten sets at 60 °C.

### Calculated optical properties

**Figure 1:**
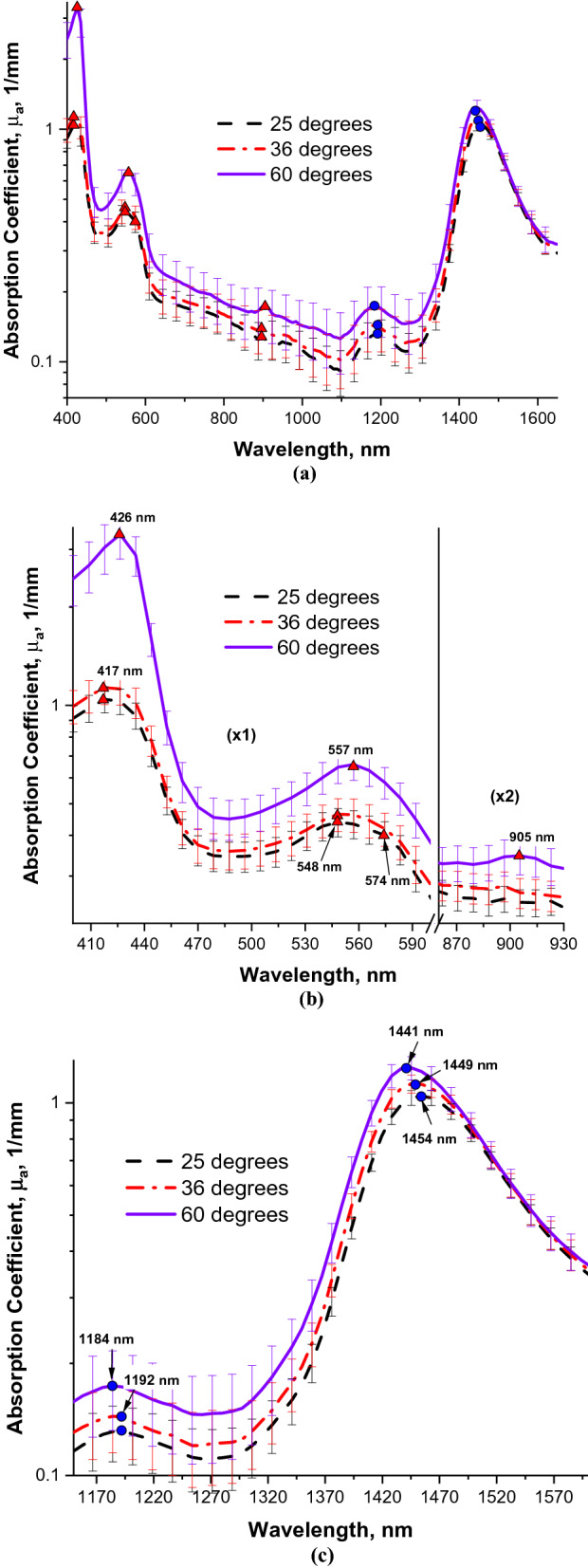
Absorption coefficients between (**a**) 400–1650 nm, (**b**) 400–930 nm (note the scale change in the NIR region), and (**c**) 1150–1600 nm. Dashes—25 °C. Dashes and dots—36 °C. Solid line—60 °C. Bars—standard errors. Red triangles—maxima of hemoglobin peaks. Blue circles—maxima of water peaks.

In Fig. 1, the averaged absorption coefficients (*µ*_*a*_) in the spectral range between 400 and 1650 nm are presented. Figure 1a demonstrates that at all the temperatures investigated, absorption of mouse ear tissue is dominated by hemoglobin in the spectral range from 400 to 950 nm and by water in the spectral range from 1150 to 1650 nm. Wavelength dependence of the absorption coefficients at 25 °C and at 36 °C is similar both qualitatively and quantitatively. In contrast, absorption coefficients at 60 °C are significantly higher than those at the lower temperatures. There are also clearly pronounced spectrally dependent differences. Figure 1b shows a magnified view of the absorption bands between 400 and 930 nm. It can be readily appreciated that Soret absorption band of hemoglobin exhibits a red shift from 417 nm at the lower temperatures to 426 nm at 60 °C. Even though ketamine-xylazine anesthetic reduces hemoglobin oxygenation^28^, double absorption peak of oxyhemoglobin between 545 and 575 nm is discernible at the lower temperatures. It is replaced by a single peak of deoxy-hemoglobin at approximately 557 nm at 60 °C. In addition, there appears a deoxy-hemoglobin absorption band around 905 nm. Figure 1c displays a magnified view of the absorption bands between 1150 and 1600 nm with two water absorption peaks in the proximity of 1200 nm and 1450 nm. Both peaks exhibit considerable blue shifts as tissue temperature increased to 60 °C. Specifically, the water absorption peak shifts from 1192 nm at 25 °C and 36 °C to 1184 nm at 60 °C, whereas the absorption peak located at 1454 nm at 25 °C shifts to 1449 nm at 36 °C and to 1441 nm at 60 °C. Statistical analysis confirmed that the blue shifts in water absorption as temperature increased from 25 to 60 °C were highly significant (*P* < 0.0001).

**Figure 2:**
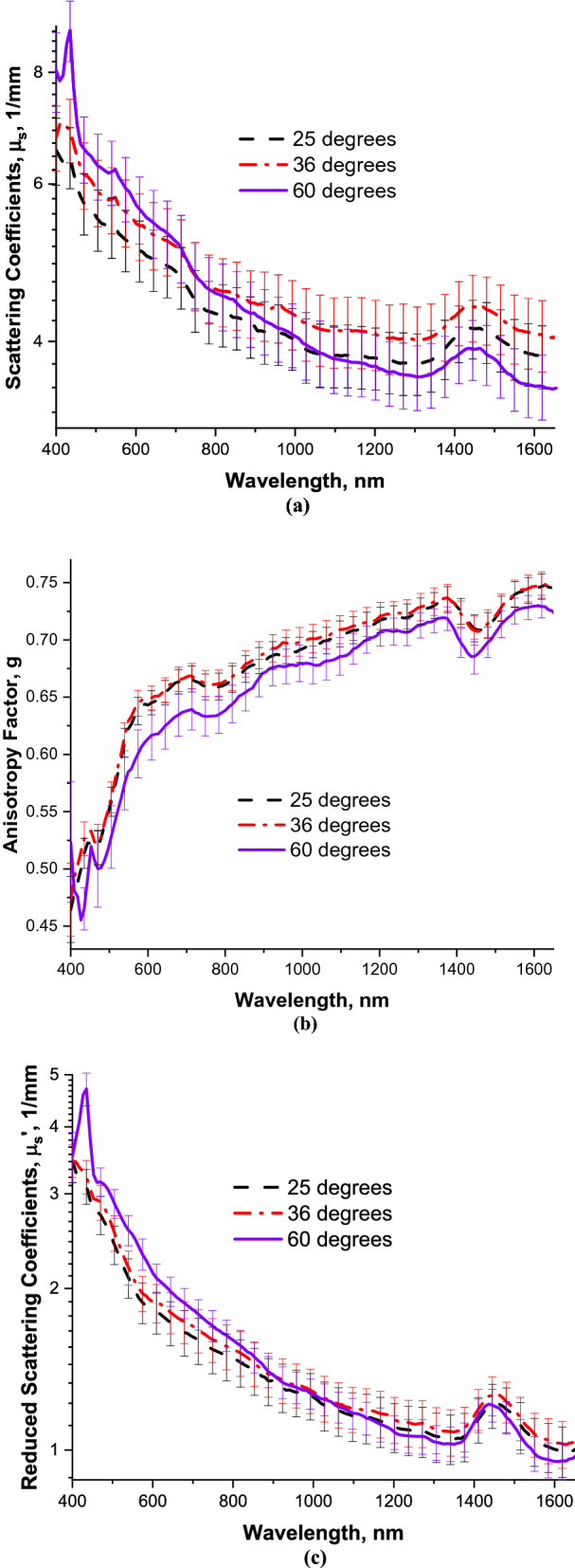
(**a**) Scattering coefficients between 400–1650 nm. (**b**) Anisotropy factors between 400–1650 nm. (**c**) Reduced scattering coefficients between 400–1650 nm. Dashes—25 °C. Dashes and dots—36 °C. Solid line—60 °C. Bars—standard errors.

Calculated scattering coefficients (*µ*_*s*_), anisotropy factors (g), and reduced scattering coefficients (*µ*_*s*_*′*), at all the temperatures investigated are shown in Fig. 2. Scattering coefficients (Fig. 2a) were found to decrease with increasing wavelength at each temperature, with stronger spectral dependence at 60 °C, as compared to 25 °C and 36 °C. A much steeper slope of the scattering coefficient versus wavelength graph at 60 °C may be explained by tissue coagulation that causes destruction of the larger scatterers, such as erythrocytes, as well as homogenization of the blood vessel walls and collagen bundles in the dermis. Accordingly, the anisotropy factors, displayed in Fig. 2b, are slightly lower at 60 °C. Similar to the scattering coefficients, reduced scattering coefficients presented in Fig. 2c demonstrate steeper gradient at 60 °C, as compared to those observed at 25 or 36 °C.

### Confocal imaging

**Figure 3:**
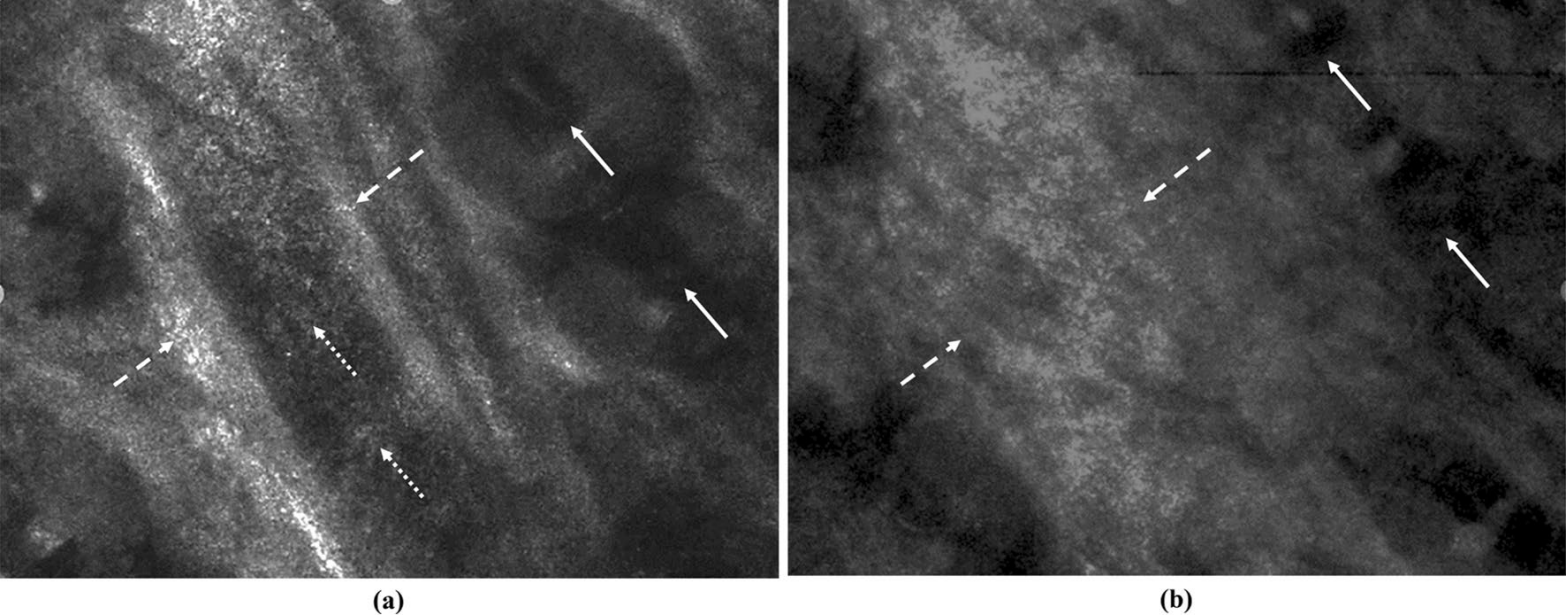
Representative reflectance confocal images of in vivo mouse ear tissue at (**a**) 25 °C and (**b**) 60 °C. Confocal videos of in vivo mouse ear tissue at 25 °C, and heated to 60 °C, are shown in Supplemental Videos S1 and S2, respectively. Dashed arrows and dotted arrows point to a vascular wall and erythrocytes inside the vessel, respectively. Solid arrows points to a hair follicle. FOV = 750 µm × 560 µm.

Reflectance confocal videos of mouse ear were recorded in vivo and used to monitor temperature dependent morphological changes as the temperature was increasing from 25 °C to 60 °C. Two image frames acquired at 25 °C and 60 °C are shown in Fig. 3a,b, respectively. Skin structures and appendages, such as hair follicles (solid arrows), and blood vessel (dashed arrows), can be seen on the background of dermal collagen in Fig. 3a. Highly scattering erythrocytes (dotted arrows) can be discerned flowing within a blood vessel. Corresponding video, presented in Supplementary Video S1, allows for a better visualization of the blood flow. The shaft of an adjacent hair follicle appears dark due to lack of melanin in albino mice. Figure 3b shows the same region at a temperature of 60 °C. At this temperature, tissues coagulate and shrink. It has been shown that coagulation process is accompanied by collagen swelling, homogenization and collapse of the blood vessel walls, and destruction of the erythrocytes^15^. All these changes are evident in the image. Reflectance from swollen collagen increases, hair follicles shrink, and the blood vessel, present in Fig. 3a, disappears. The video showing morphological changes of the tissue during its heating from 25 to 60 °C can be viewed in Supplementary Video S2.

## Discussion

Clinical adoption and optimization of diagnostic, therapeutic, and surgical applications of light require understanding of its propagation and interaction with target tissues. Therefore, optical properties of biotissues, such as absorption coefficients (*µ*_*a*_), scattering coefficients (*µ*_*s*_), and anisotropy factors (g), have been studied extensively in recent years^29–37^. Due to logistical challenges, most investigations of optical properties have been performed ex vivo^37–41^*.* Alternatively, in the minority of studies, spatially resolved diffuse reflectance of remitted light was measured to determine absorption and reduced scattering coefficients using the diffusion approximation of light transport in turbid media^24–27^. To the best of our knowledge, this is the first comprehensive study that reports and quantifies temperature induced changes of tissue optical properties in vivo in the spectral range of 400–1650 nm using integrating sphere spectrophotometry and inverse Monte Carlo data processing. Still, several groups investigated temperature induced changes of skin, blood and water. For example, Helfmann et al.^42^, determined absorption, scattering, and anisotropy of oxygenated and deoxygenated blood using a double integrating sphere system and an Inverse Monte Carlo method. They report absorption peaks located at 415 nm, 540 nm, and 575 nm for oxygenated blood, and 430 nm and 550 nm for deoxygenated blood. These spectral differences between oxygenated and deoxygenated blood match differences found between 25 and 60 °C absorption spectra found in the present study. Jia et al.^43^, measured the absorbance of blood in the spectral range of 500–650 nm while samples were heated to 60–80 °C. They reported an increased contribution from deoxyhemoglobin to absorbance measurements when heated to 60–80 °C. Findings from both Helfmann et al.^42^, and Jia et al.^43^, confirm the deoxygenation of blood with increasing temperature found in the present study. Nilsson et al.^44^, determined the optical properties of whole blood, heated to 25–55 °C, at 633 nm. In agreement with our study, they found an increase in both scattering and absorption, as well as slight decrease of anisotropy factor of heated blood at 633 nm. Barton et al.^45^, determined that pulsed laser illumination of hamster dorsal skin flap caused hemoglobin deoxygenation, blood coagulation, as well as blood vessel constriction due to tissue heating. They have also noted significant increase of tissue absorption coefficients. These results are concordant with our findings. Barton et al.^45^ also reported the formation of methemoglobin after laser illumination. Methemoglobin was not detected in the present study at 60 °C. This discrepancy may be explained by the discovery of Jia et al.^43^, that measurable amounts of methemoglobin start to form as blood temperatures reach 70 °C.

Laufer et al.^17^, measured the temperature induced changes in absorption and reduced scattering using an integrating sphere system and an Inverse Monte Carlo method. They report an increase in reduced scattering found in ex vivo human dermis as tissue temperature increased from 25 to 40 °C in the 600–1000 nm spectral range. This increase in reduced scattering agrees with the findings in the present study. Laufer et al.^17^, reported no significant changes in tissue absorption in this spectral range. Similarly, our study revealed that temperature induced changes in absorption coefficients in the spectral range between 600 and 1000 nm are minimal.

Several studies investigated temperature induced blue shifts of water absorption^46–52^. In particular, Collins^46^ measured the absorbance of water samples between 0 and 95 °C and reported a 40 nm and a 20 nm blue shift of the absorption peaks in the vicinity of 1200 nm and 1450 nm, respectively. These shifts in the absorption spectra qualitatively agree with those found in this present study. However, Collins^46^ did not observe an increase in amplitude of the 1450 nm peak found in our study. This increase can be explained by temperature induced edema, which does not occur in pure water samples.

Overall, the comparison of the results obtained in this study shows that our data are in qualitative agreement with the trends reported in the literature for ex vivo and in vivo tissues, when the latter are available. The discrepancies as well as the variations within the values reported in the literature are most probably due to the variations in experimental conditions, as well as differences in theoretical models and tissue treatment techniques employed in the studies. In conclusion, we have investigated the temperature induced changes of the mouse ear skin optical properties in vivo between 400 and 1650 nm in the temperature range between 25 °C and 60 °C. Our results demonstrate that the major differences in absorption are caused by deoxygenation of blood and heating of water, while the major differences in scattering are caused by blood and collagen coagulation. Reflectance confocal microscopy confirms these findings. Imaging reveals tissue shrinkage during its heating from 25 to 60 °C. This shrinkage leads to the increased concentration of chromophores and explains significant rise of absorption coefficients at 60 °C as compared to 25 °C and 36 °C. The results of this study may provide a reliable foundation for dosimetry of therapeutic and diagnostic clinical procedures.

## Methods

All methods were carried out in accordance with relevant guidelines and regulations.

### Animal handling

All procedures involving animals were performed under a protocol approved by the Massachusetts General Hospital Subcommittee on Research Animal Care and followed the ARRIVE guidelines for reporting animal experiments. The BALB/c mice used in this study were obtained from Charles River Breeding Laboratory (Wilmington, MA).

Prior to the spectroscopic experiments, anesthesia was induced using an intraperitoneal injection of ketamine-xylazine mix (10:1) at a dose of 1 µL/g body weight. The anesthesia was maintained for up to 30 min, providing adequate time to perform the measurements. Mice were sacrificed immediately following the final measurements.

### Integrating sphere spectrophotometry

An in-house built single integrating sphere spectrophotometer, described in detail elsewhere^33^, was used for acquiring total transmittance, diffuse transmittance, and diffuse reflectance over the spectral range of 400–1650 nm. Spectroscopic measurements were performed at 25 °C, 36 °C, and 60 °C. A halogen lamp (HL-2000, 360–2000 nm, Ocean Optics, Dunedin, FL) was used as the light source. Intensity fluctuations of the lamp did not exceed 0.1% over 6 h. Two grating spectrophotometers, an HR2000 spectrometer (Ocean Optics, Dunedin, FL) and an EPP2000-NIR InGaAs spectrometer (StellarNet, Tampa, FL) simultaneously attached to the two auxiliary ports of the integrating sphere (4P-GPS-033-SL, Labsphere, North Sutton, NH) were used for measuring spectral responses in the 400–980 nm and the 900–1650 nm range, respectively.

For total and diffuse transmittance measurements, light emitted from a halogen lamp was focused on mouse ears that were attached to the entrance port of the integrating sphere. Transmittance through air was used as reference. The diameter of the focused beam was 1.9 mm and the diameter of the entrance port was 5 mm. For measuring total transmittance, the exit port of the integrating sphere was closed. For measuring diffuse transmittance, the exit port was open to allow un-scattered light to escape. Collimated transmittance of the mouse ear was calculated by subtracting diffuse transmittance from the total transmittance signal for each wavelength of the spectral range investigated.

For diffuse reflectance measurements, light was focused on the mouse ears attached to the exit port of the integrating sphere. A 99% spectralon diffuse reflectance standard (Labsphere Inc., North Sutton, NH) was used as a reference. The focal spot size was 1.9 mm, the diameter of the exit port was 5 mm, and the entrance port of the integrating sphere had a diameter of 25.4 mm.

### Data processing technique

An inverse Monte Carlo algorithm, described in detail previously^53^, was used to determine the optical properties from the measured quantities. The Monte Carlo (MC) technique took into account the exact geometrical and optical parameters of the experimental arrangement, the three-layer structure of the object under investigation (sapphire glass—mouse ear—sapphire glass) and losses of light at the edges of the ear. The MC technique was incorporated as a forward procedure into quasi-Newton inverse algorithm. The inverse algorithm employed Broyden update formula to reduce the number of the forward model calls and the “trust region” approach to achieve proximity of the solution even when the initial approximation was poor^54^. The inverse technique allowed determination of absorption coefficients, scattering coefficients and anisotropy factors from the measurements of total transmittance, collimated transmittance, and diffuse reflectance, while parameters of the phase function were pre-set. The anisotropy factors were determined under the assumption of the Henyey–Greenstein scattering phase function^55^.

### Temperature control system

**Figure 4:**
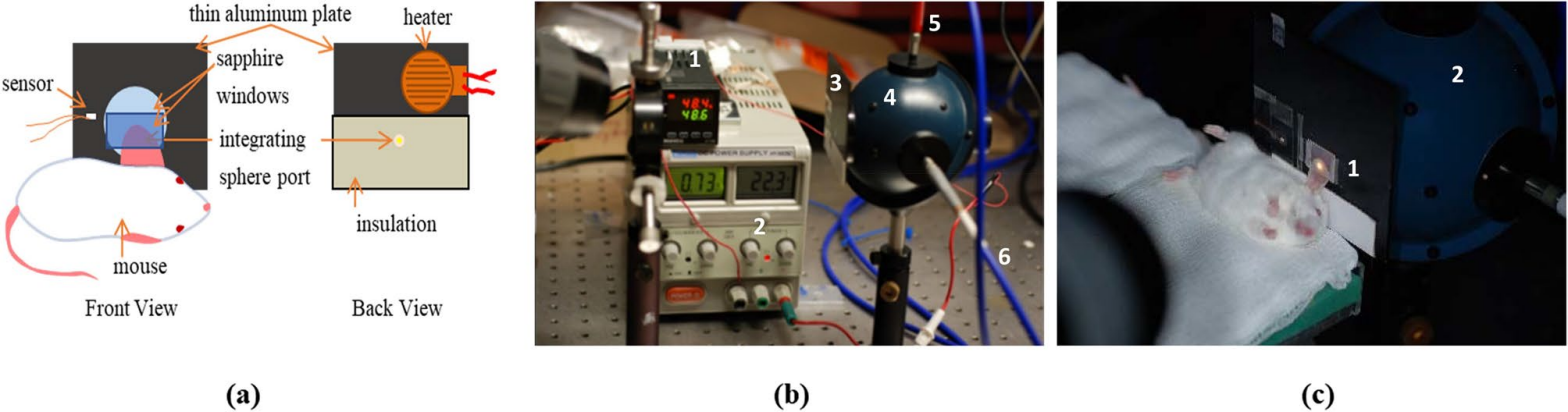
Lay-out of the integrating sphere experiments. (**a**) Diagram of the heating apparatus that was used to heat and maintain the desired tissue temperature while spectroscopic measurements were performed. Insulation between the aluminum plate and the integrating sphere, as well as between the aluminum plate and the mouse body prevented unnecessary heating. (**b**) Picture of integrating sphere system with heating apparatus. Temperature controller (1) was attached to a power supply (2) and aluminum plate (3). Controller (1) triggered external power for heating or cooling. Aluminum plate was attached to the sample port of the integrating sphere (4). Light collected by the integrating sphere passed through two optical fibers (5 and 6) coupled to the visible and NIR spectrometers. (**c**) Picture of total transmittance data acquisition. The mouse ear, sandwiched between two sapphire windows attached to the aluminum plate (1), was heated by the heater and the temperature was controlled by the temperature controller. Light transmitted through the ear was collected by the integrating sphere (2).

The temperature control system is depicted in Fig. 4a. Mouse ears were sandwiched between two sapphire windows and placed on top of an aluminum plate with the hole of the size of the entrance / exit port of the integrating sphere (diameter of 5 mm). This hole in the aluminum plate enabled light to pass through and enter the integrating sphere. A heater (HK5541R30, Minco, Minneapolis, MN) was attached to the aluminum plate and powered by an external power supply (Tekpower HY3005D, Tekpower, Montclair, CA). The heater and power supply were connected to a temperature control unit (CT16A2080-948, Minco, Minneapolis, MN), and a temperature sensor was attached to the aluminum plate. The aluminum plate reached the desired temperature within 30 s of heating and the desired temperature was maintained within ± 0.1 °C. Photographs of the integrating sphere system with heating apparatus are displayed in Fig. 4b,c. In addition, the temperature was concurrently monitored using a thermal camera (ThermaCAM PM390, FLIR Systems, Wilsonville, OR). Temperature settings of 25 °C, 36 °C, and 60 °C were used during collection of the total transmittance, diffuse transmittance, and diffuse reflectance data.

### Confocal microscopy

**Figure 5:**
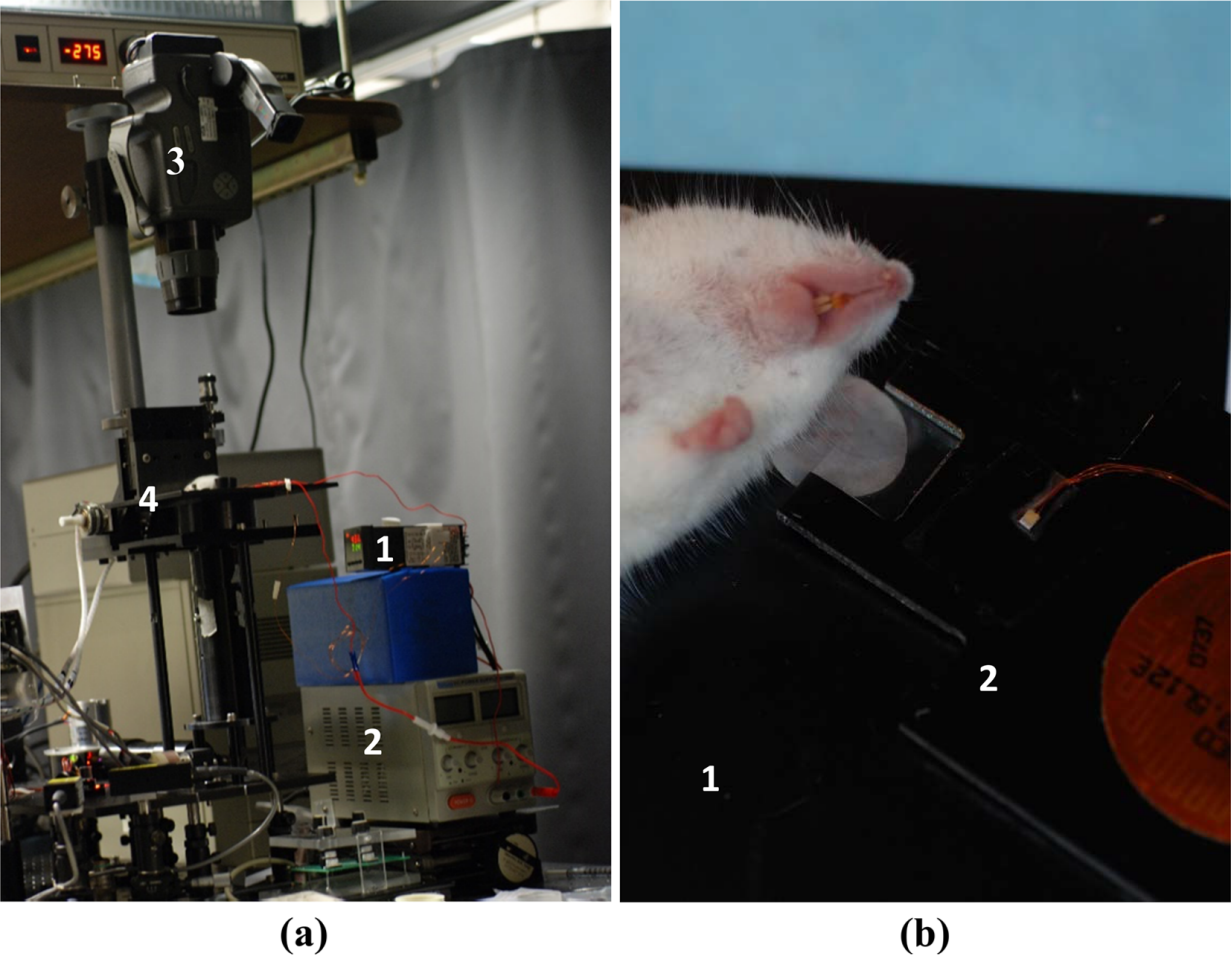
Imaging experiments lay-out. (**a**) Reflectance confocal microscope with heating apparatus and thermal camera. External temperature controller (1) and power supply (2) were used to heat and maintain the desired tissue temperature. Thermal camera (3) was positioned above the sample stage (4) to provide independent temperature monitoring. (**b**) Picture of mouse ear positioning on the sample stage (1) of the confocal microscopy system. Ear tissue was heated using the same heating apparatus (2) as for the integrating sphere measurements.

To reveal structural and functional changes in the mouse ear during heating, we monitored the heating process using a commercial confocal microscope (Vivascope 2000, Lucid Inc., Rochester, New York) (Fig. 5a). A detailed description of the confocal system is available in a previous publication^56^. In short, illumination was provided by 830 nm diode laser (Micro Laser Systems Inc., Garden Grove, CA). Laser light was focused onto the sample by a 20x/NA 0.75 water-immersion objective lens (Nikon Inc., Tokyo, Japan). The imager provided lateral and axial resolution of 1.5 µm and 5 µm, respectively. The imaging depth was approximately 100 µm with a field of view of 800 × 600 µm^2^. The mice were placed supine on the inverted sample stage (Fig. 5b). Confocal images and videos were acquired as mouse ears were heated from 25 °C to 60 °C using the temperature control system described above. For validation purposes, temperature was concurrently monitored using a thermal camera (ThermaCAM PM390, FLIR Systems, Wilsonville, OR).

### Statistical analysis

The optical data were evaluated statistically to obtain least squares estimates of mean gradient (slopes of scattering and reduced scattering) or shifts of absorption maxima, along with corresponding standard errors, for each temperature group. A mixed effects model^57^ was used to assess differences between gradients and extremum shifts observed at 25 °C/60 °C and 36 °C/60 °C with *P* ≤ 0.05 considered significant.

### Ethical approval

All procedures involving animals were performed under a protocol approved by the Massachusetts General Hospital Subcommittee on Research Animal Care. All procedures involving animals were reported following the ARRIVE guidelines.

## Supplementary Information

Supplementary video 1.

Supplementary video 2.

Supplementary Information.

## Data Availability

Datasets related to this article can be obtained from the corresponding author.
