# Supplementary material for: Temperature induced changes in the optical properties of skin in vivo
Source: Sci Rep. 2021 Jan 12;11:754. doi: 10.1038/s41598-020-80254-9 (PMC7803738; doi:10.1038/s41598-020-80254-9)

## Slide 1
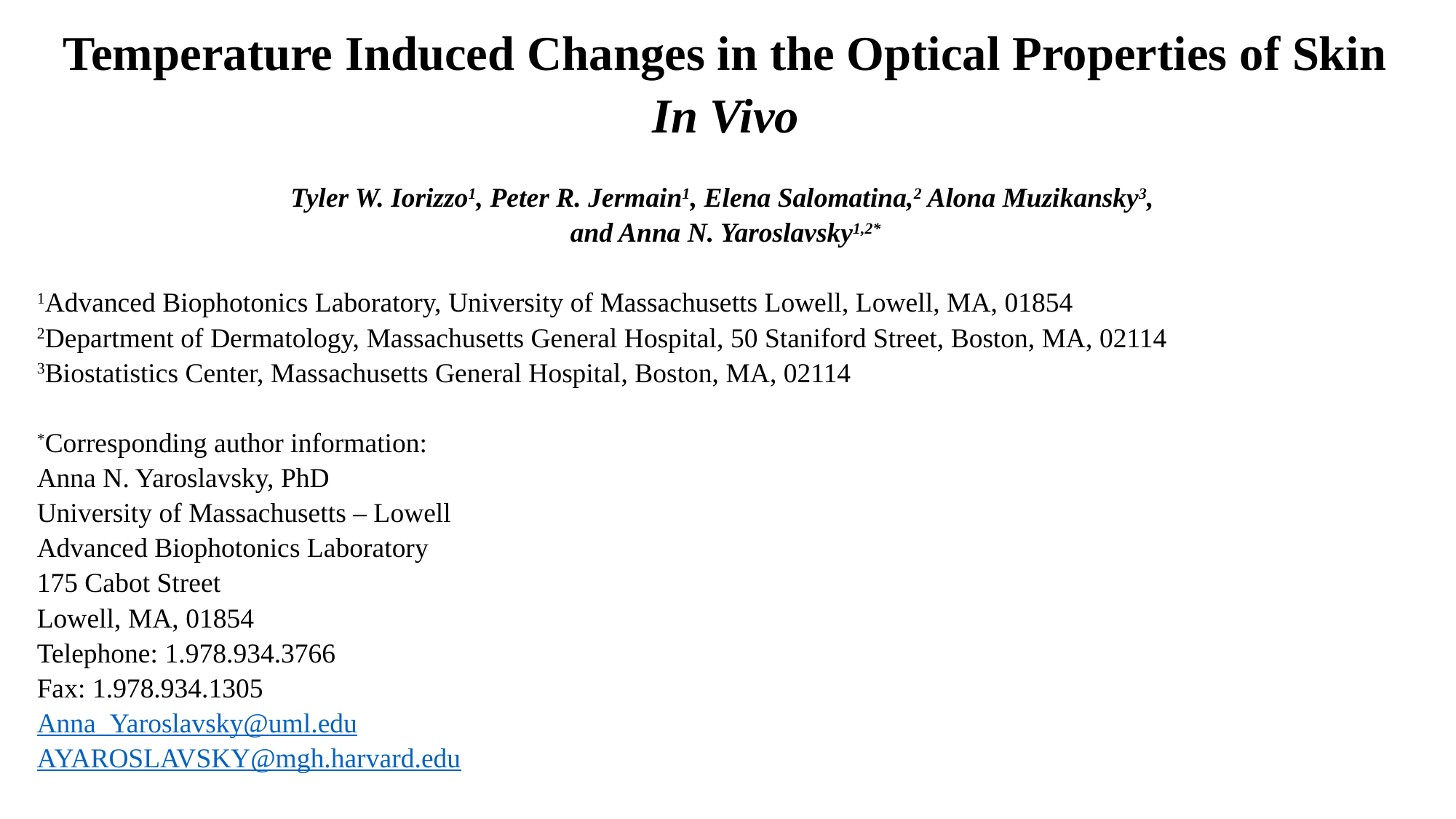

Temperature Induced Changes in the Optical Properties of Skin In Vivo
Tyler W. Iorizzo1, Peter R. Jermain1, Elena Salomatina,2 Alona Muzikansky3,
and Anna N. Yaroslavsky1,2*
1Advanced Biophotonics Laboratory, University of Massachusetts Lowell, Lowell, MA, 01854
2Department of Dermatology, Massachusetts General Hospital, 50 Staniford Street, Boston, MA, 02114
3Biostatistics Center, Massachusetts General Hospital, Boston, MA, 02114
*Corresponding author information:
Anna N. Yaroslavsky, PhD
University of Massachusetts – Lowell
Advanced Biophotonics Laboratory
175 Cabot Street
Lowell, MA, 01854
Telephone: 1.978.934.3766
Fax: 1.978.934.1305
Anna_Yaroslavsky@uml.edu
AYAROSLAVSKY@mgh.harvard.edu

## Slide 2
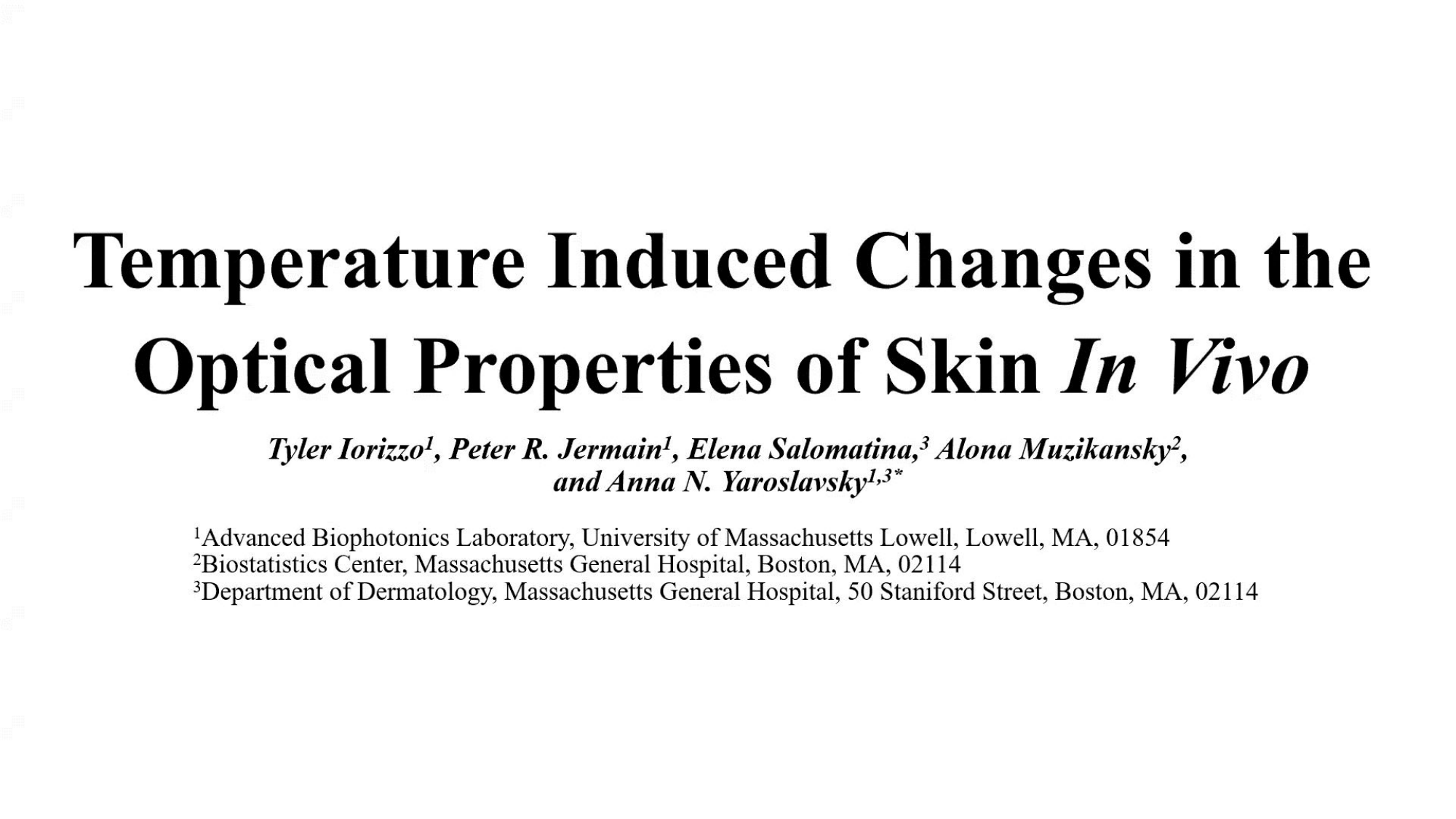

## Slide 3
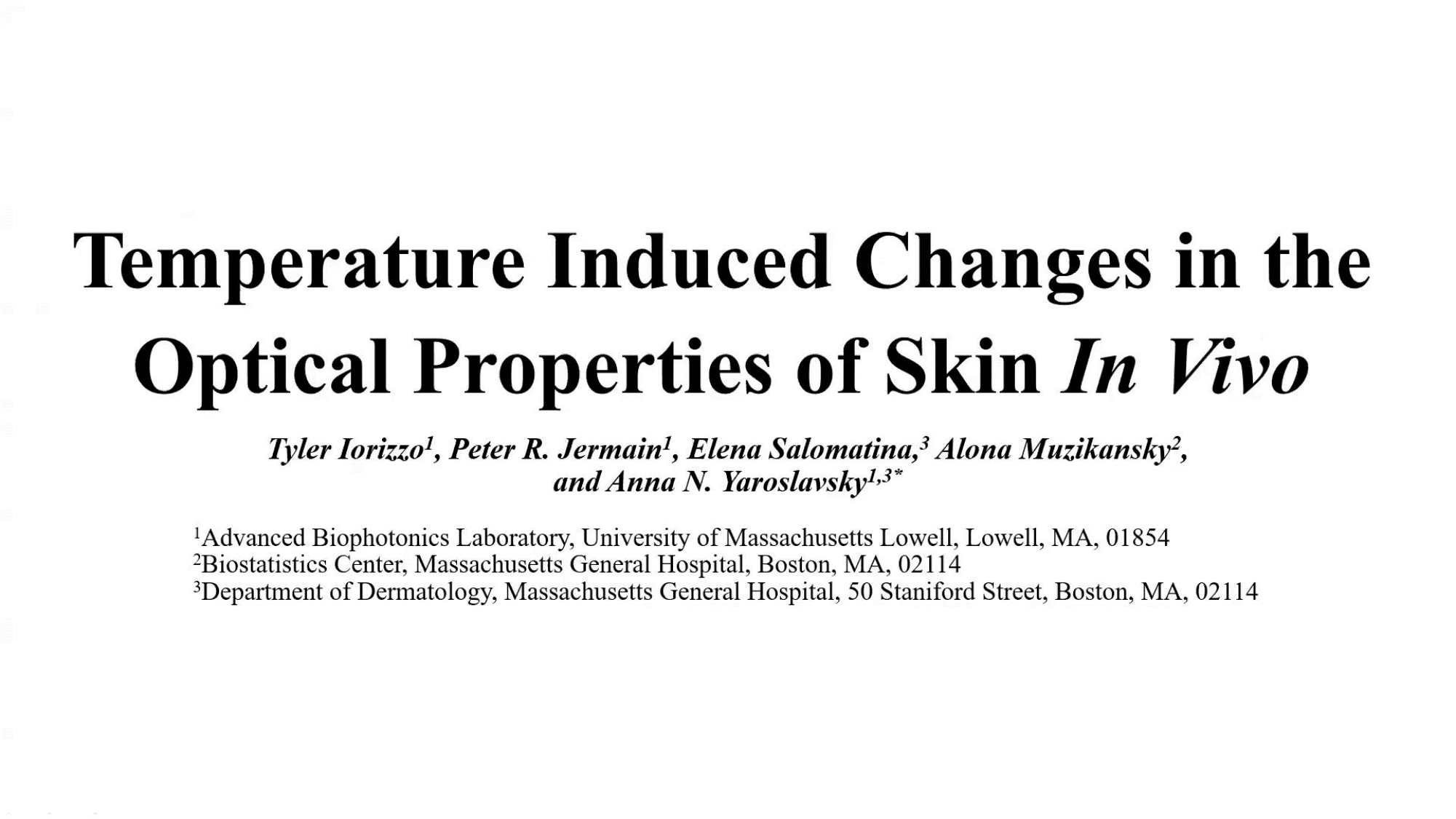

Supplement: Supplementary file 3 — Supplementary Information. [file 41598_2020_80254_MOESM3_ESM.pptx]
